# LIPAD (LRRK2/Luebeck International Parkinson's Disease) Study Protocol: Deep Phenotyping of an International Genetic Cohort

**DOI:** 10.3389/fneur.2021.710572

**Published:** 2021-08-09

**Authors:** Tatiana Usnich, Eva-Juliane Vollstedt, Nathalie Schell, Volha Skrahina, Xenia Bogdanovic, Hanaa Gaber, Toni M. Förster, Andreas Heuer, Natalia Koleva-Alazeh, Ilona Csoti, Ayse Nazli Basak, Sibel Ertan, Gencer Genc, Peter Bauer, Katja Lohmann, Anne Grünewald, Emma L. Schymanski, Joanne Trinh, Susen Schaake, Daniela Berg, Doreen Gruber, Stuart H. Isaacson, Andrea A. Kühn, Brit Mollenhauer, David J. Pedrosa, Kathrin Reetz, Esther M. Sammler, Enza Maria Valente, Franco Valzania, Jens Volkmann, Simone Zittel, Norbert Brüggemann, Meike Kasten, Arndt Rolfs, Christine Klein, The LIPAD Study Group

**Affiliations:** ^1^Institute of Neurogenetics, University of Lübeck, Lübeck, Germany; ^2^CENTOGENE GmbH, Rostock, Germany; ^3^Gertrudis Clinic Biskirchen, Parkinson-Center, Leun, Germany; ^4^Neurodegeneration Research Laboratory, Suna and Inan Kirac Foundation, Koç University Translational Medicine Research Center, Koç University School of Medicine, Istanbul, Turkey; ^5^Department of Neurology, Koç University School of Medicine, Istanbul, Turkey; ^6^Sişli Etfal Training and Research Hospital, Istanbul, Turkey; ^7^Luxembourg Centre for Systems Biomedicine, University of Luxembourg, Esch-sur-Alzette, Luxembourg; ^8^Department of Neurology, University Hospital Schleswig-Holstein, Kiel, Germany; ^9^Neurologisches Fachkrankenhaus Für Bewegungsstörungen/Parkinson, Beelitz, Germany; ^10^Parkinson's Disease and Movement Disorder Center of Boca Raton, Boca Raton, FL, United States; ^11^Department of Neurology and Experimental Neurology, Charité Medical University Berlin, Berlin, Germany; ^12^Paracelsus-Elena-Klinik, Kassel, Germany; ^13^Department of Neurology, University Hospital of Gießen and Marburg, Marburg, Germany; ^14^Department of Neurology, University Hospital Rheinisch-Westfälische Technische Hochschule Aachen, Aachen, Germany; ^15^Medical Research Council Protein Phosphorylation and Ubiquitylation Unit and Molecular and Clinical Medicine, Ninewells Hospital and Medical School, University of Dundee, Dundee, United Kingdom; ^16^Department of Molecular Medicine, University of Pavia and Istituto di Ricovero e Cura a Carattere Scientifico Mondino Foundation, Pavia, Italy; ^17^Neurology Unit, Azienda USL – IRCCS di Reggio Emilia, Reggio Emilia, Italy; ^18^Department of Neurology, University Hospital Würzburg, Würzburg, Germany; ^19^Department of Neurology, University Medical Center Hamburg-Eppendorf, Hamburg, Germany; ^20^Department of Neurology, University of Lübeck, Lübeck, Germany; ^21^Department of Psychiatry and Psychotherapy, University of Lübeck, Lübeck, Germany

**Keywords:** Parkinson's disease, LRRK2, GBA, clinical study, genetic cohort

## Abstract

**Background:** Pathogenic variants in the Leucine-rich repeat kinase 2 (*LRRK2)* gene are the most common known monogenic cause of Parkinson's disease (PD). *LRRK2*-linked PD is clinically indistinguishable from idiopathic PD and inherited in an autosomal dominant fashion with reduced penetrance and variable expressivity that differ across ethnicities and geographic regions.

**Objective:** To systematically assess clinical signs and symptoms including non-motor features, comorbidities, medication and environmental factors in PD patients, unaffected *LRRK2* pathogenic variant carriers, and controls. A further focus is to enable the investigation of modifiers of penetrance and expressivity of *LRRK2* pathogenic variants using genetic and environmental data.

**Methods:** Eligible participants are invited for a personal or online examination which comprises completion of a detailed eCRF and collection of blood samples (to obtain DNA, RNA, serum/plasma, immune cells), urine as well as household dust. We plan to enroll 1,000 participants internationally: 300 with *LRRK2*-linked PD, 200 with *LRRK2* pathogenic variants but without PD, 100 PD patients with pathogenic variants in the *GBA* or *PRKN* genes, 200 patients with idiopathic PD, and 200 healthy persons without pathogenic variants.

**Results:** The eCRF consists of an investigator-rated (1 h) and a self-rated (1.5 h) part. The first part includes the Movement Disorder Society Unified Parkinson's Disease Rating, Hoehn &Yahr, and Schwab & England Scales, the Brief Smell Identification Test, and Montreal Cognitive Assessment. The self-rating part consists of a PD risk factor, food frequency, autonomic dysfunction, and quality of life questionnaires, the Pittsburgh Sleep Quality Inventory, and the Epworth Sleepiness as well as the Hospital Anxiety and Depression Scales. The first 15 centers have been initiated and the first 150 participants enrolled (as of March 25th, 2021).

**Conclusions:** LIPAD is a large-scale international scientific effort focusing on deep phenotyping of *LRRK2*-linked PD and healthy pathogenic variant carriers, including the comparison with additional relatively frequent genetic forms of PD, with a future perspective to identify genetic and environmental modifiers of penetrance and expressivity

**Clinical Trial Registration:**ClinicalTrials.gov, NCT04214509.

## Introduction

Pathogenic variants in the Leucine-rich repeat kinase 2 (*LRRK2*) gene are the most common known monogenic cause of Parkinson's disease (PD). First identified in 2004, *LRRK2* pathogenic variants account for up to ~40% of all PD cases in selected populations, e.g., North African Berber (1). In the PD phenotype-genotype database MDSGene (www.mdsgene.org), ~700 individual *LRRK2* pathogenic variant carriers are listed, but clinical information apart from cardinal signs of PD is overall scarce (1).

The majority of the reported *LRRK2* pathogenic variant-positive PD patients are of European descent (63%), whereas all other ethnicities comprise ~10% or fewer patients of described pathogenic variant carriers despite clusters in the Ashkenazi Jewish (AJ) and Arab Berber populations (1, 2). Definitely pathogenic variants identified in *LRRK2* include p.G2019S, p.R1441C/G/H, p.N1437H, p.Y1699C, and, p.I2020T. Of these, the p.G2019S pathogenic variant is the most common with an estimated frequency of 1% in sporadic and 4% in hereditary PD cases worldwide (3). However, data on many populations are still missing.

*LRRK2*-linked PD is inherited in an autosomal dominant fashion with reduced, age-dependent penetrance and variable expressivity that differ across ethnicities and geographic regions, indicating that ancestral background or environmental factors contribute to the manifestation and expressivity of pathogenic *LRRK2* variants. For example, at the age of 60 years, 60% of Tunisian p.G2019S pathogenic variant carriers manifest PD, compared with only 20% of the Norwegian carriers of the same pathogenic variant (4). Recent studies have suggested mitochondrial involvement in the penetrance of *LRRK2*-linked PD (5, 6). Environmental factors can also influence penetrance, as recently found in the Tunisian Arab Berber population, where tobacco and black tea use delayed age at onset in *LRRK2* pathogenic variant carriers (7).

*LRRK2*-linked PD is clinically indistinguishable from idiopathic PD, i.e., PD of unknown cause, although the disease course appears to be milder with regard to motor and cognitive functions (8). In this comparatively small subgroup of PD patients with a known etiology, valuable clues can be garnered toward pathophysiological mechanisms and targeted treatments, which may be generalized to idiopathic PD. Currently, there are three substances in clinical trial specifically targeting *LRRK2*: the LRRK2 kinase inhibitors DNL151 (NCT04557800) and DNL201 (NCT04551534) from Denali and the antisense oligonucleotide BIIB094 (NCT03976349) from Biogen (2). However, no genetic testing guidelines and no clinical trial-ready cohorts exist to date.

To identify *LRRK2* pathogenic variant carriers among 10,000 PD patients from 15 countries and to establish a clinical trial-ready cohort, the ROPAD study was initiated (9). The ROPAD study performs genetic testing of pathogenic variants in the *LRRK2* and *GBA* genes and, if negative, carries out genetic screening of 68 genes linked to PD or related movement disorders. Out of the first 1,288 participants who underwent genetic testing in ROPAD, 40 (3.1%) harbored pathogenic or likely pathogenic *LRRK2* variants, 109 (8.5%) harbored *GBA* variants, three (0.2%) had alterations in both genes (9). The ROPAD study is still ongoing and continues to identify *LRRK2* pathogenic variant carriers internationally. It gathers only basic clinical data: age at examination, age at onset, cardinal PD symptoms, and 12/33 items of MDS-UPDRS Part III and, thus, does not allow a systematic in-depth characterization of *LRRK2* pathogenic variant carriers.

Recently published data from the currently largest multinational prospective study that includes *LRRK2* pathogenic variant carriers, i.e., the Parkinson's Progression Markers Initiative (PPMI), provide more phenotypic details on *LRRK2* pathogenic variant carriers across international sites (6). However, as the focus of the PPMI study is predominantly on biomarkers, it does not cover environmental factors in-depth, and only includes participants with a PD diagnosis 2 years or less before enrolment who are untreated with PD medication. There remains a gap in combining phenotypic, genetic, and environmental data on *LRRK2* pathogenic variant carriers internationally, regardless of the onset of signs and symptoms and their medication status, that LIPAD is aiming to close. The objectives of the LIPAD study are (1) to provide a systematic characterization of PD patients and unaffected carriers with pathogenic variants in the *LRRK2* gene; and (2) to enable the investigation of modifiers of penetrance of *LRRK2* pathogenic variants using genetic and environmental data.

Here, we describe a protocol and a feasibility study of the first 150 participants and give a brief overview of nested studies within LIPAD. Of note, the LIPAD protocol has also been adapted to take place online due to COVID19 contact restriction measures.

## Patients and Methods

### General Setting

**Figure 1:**
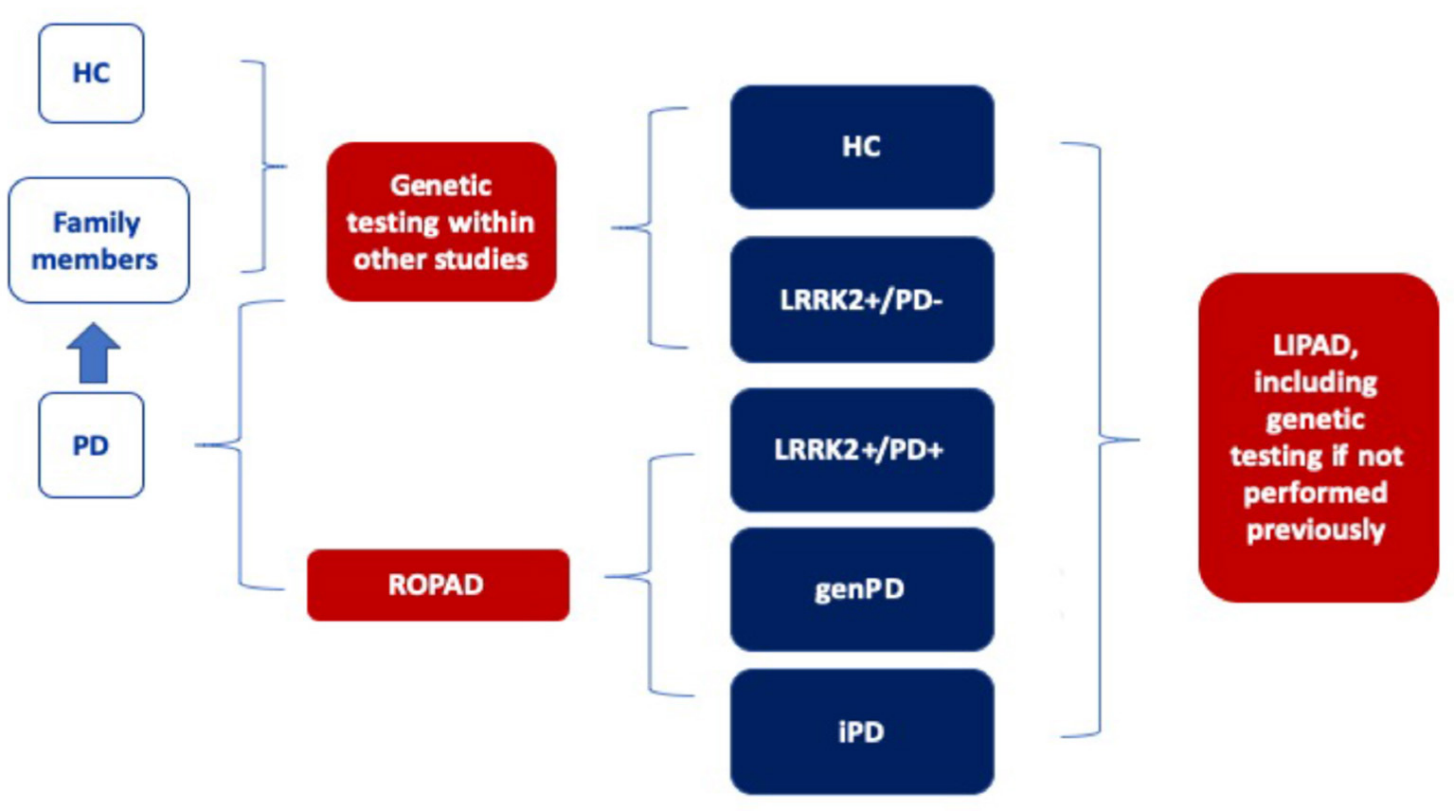
Paths of enrolment into the LIPAD study. *LRRK2*+/PD+: PD patients carrying a pathogenic LRRK2 variant, *LRRK2*+/PD-: unaffected carriers of pathogenic *LRRK2* variants, genPD: PD patients with pathogenic variants in PD genes other than *LRRK2, iPD; LRRK2-/PD*+*:* patients with idiopathic PD from the same populations, HC: healthy persons without pathogenic variants.

We aim to build the LIPAD cohort consisting of 1,000 participants internationally: 300 of them with *LRRK2*-PD (*LRRK2*+/PD+), 200 with pathogenic *LRRK2* variants but without PD (*LRRK2*+/PD-), 100 PD patients with pathogenic variants in PD genes other than *LRRK2* (genPD), 200 patients with idiopathic PD from the same populations (iPD; *LRRK2*-/PD+), and 200 healthy persons without pathogenic variants (HC). The majority of participants will be enrolled after genetic analysis within ROPAD (Figure 1). Extrapolating from the ROPAD findings, we expect to have a sufficient number of participants in the *LRRK2*+/ PD+, iPD, and genPD groups. Further, family members of pathogenic *LRRK2* variant carriers will be asked to participate in the LIPAD study and will be genetically tested for the familial pathogenic *LRRK2* variant. Depending on the results, they will be assigned either to *LRRK2*+/PD- or HC group. Healthy persons without pathogenic variants will be recruited from spouses of study participants or the general population. Within an anticipated 24-month recruitment period per site, ~25 centers in 10 countries on three continents will be established. Each center will have 24 months for recruitment after initiation. The majority of the centers will be those already participating in ROPAD, however, additional centers that do not participate in ROPAD but follow *LRRK2* pathogenic variant carriers will be invited to become LIPAD sites.

### Inclusion Criteria

Recruited participants have to meet one of the following criteria: (i) clinical diagnosis of PD according to the Movement disorder Society (MDS) diagnostic criteria, (ii) first- or second-degree relative of a participant that is positive for a pathogenetic *LRRK2* variant, (iii) healthy participants with or without a family history of PD in a control group. All participants have to be above 18 years of age and have to sign an informed consent form.

Participants with the following *LRRK2* variants will be included in the *LRRK2*+ group: p.Gly2019Ser (c.6055G>A), p.Arg1441His (c.4322G>A), p.Arg1441Cys (c.4321C>T), p.Arg1441Gly (c.4321C>G), p.Asn1437His (c.4309A>C), p.Ile2020Thr (c.6059T>C), p.Tyr1699Cys (c.5096A>G).

### Study Design

Upon completion of genetic screening (e.g., in ROPAD), participants are invited for a personal examination using the three-level biomaterial protocol. During a single visit, an electronic Case Report Form (eCRF) is administered and blood samples for DNA, RNA, serum/plasma, immune cell isolation, urine, and household dust for toxicological analyses are collected.

The three-level protocol was designed to accommodate available laboratory facilities at the different sites. The details of the three levels for biomaterial sampling are listed in Supporting Information Methods S2 in Supplementary Material.

In case genetic testing has not been performed before, a dried blood spot card will be sent for DNA extraction and genetic screening, as specified in ROPAD.

There are no serious safety concerns associated with the study. Participants may experience temporary discomfort during venous blood sampling and while answering the questions. However, they are instructed that they are free to omit questions they would rather not answer. There are no health risks associated with the collection of urine and house dust.

### Data Analysis

Data analysis will be performed at the Institute of Neurogenetics (University of Luebeck, Germany). To cover the first objective, which is the systematic characterization of PD patients and unaffected carriers with pathogenic variants in the *LRRK2* gene, we will describe the frequency of all clinical signs and symptoms including non-motor signs and the most important influencing factors such as sex, disease duration, and medication. This will result in raw and corrected frequencies with 95% confidence intervals. We will use t-tests for numerical and continuous variables and chi-square tests for categorical variables at a significance level of 0.05 for the comparisons of the clinical signs and symptoms across the groups.

For aim 2, which is to investigate the modifiers of penetrance of pathogenic *LRRK2* variants using genetic and environmental data, we will examine penetrance in logistic regression models to quantify the influence of different factors impacting penetrance.

### Nested Studies

All LIPAD centers can suggest nested projects, which will be coordinated by a Scientific Advisory Board. These projects can apply for external funding, will have individual ethics approvals, and can use the ROPAD/LIPAD network. The following nested studies are planned or have started at the Institute of Neurogenetics (University of Luebeck):

#### Multimodal High-Quality Magnetic Resonance Imaging (MRI)

Penetrance of *LRRK2*-linked PD depends on the age of a given individual subject. Asymptomatic carriers may, however, already demonstrate neurodegenerative changes including hyposmia, abnormal midbrain hyperechogenicity upon transcranial sonography, pathologic dopaminergic neurotransmission, structural changes of the gray and white matter, and functional reorganization of neural networks. Although first morphological and functional brain changes in pathogenic *LRRK2* variant carriers have already been identified, the overall picture is still elusive due to the relatively low number of included subjects in most studies, ascertainment biases, and variable imaging protocols. Multimodal high-quality MRI, however, is a promising secondary endpoint for clinical trials given its wide availability, cost-effectiveness, and lack of radiation.

To reveal an MRI-based biomarker for *LRRK2*+/PD+ and prodromal *LRRK2*+/PD+, we aim to acquire the following MRI modalities:

1. Multiparameter mapping (R1, PD^*^, MT, and R2^*^) to reveal physical tissue properties.
2. Diffusion-weighted imaging for multicompartment diffusion models to reveal neuroinflammation and microstructural alterations.
3. Neuromelanin imaging to reveal substantia nigra and locus coeruleus degeneration.
4. Blood-oxygen-level-dependent functional MRI to identify changes in functional connectivity.
5. Magnetic resonance spectroscopy to depict the mechanisms of neurodegeneration, such as involvement of energy metabolism due to mitochondrial dysfunction, which may be subject to faster change than other aspects of MR-based imaging (e.g., changes in gray matter).

#### Metabolic Studies

To investigate whether metabolic changes in *LRRK2* carriers already exist before the motor manifestation or are secondary to it, we conduct the following studies:

1. Energy consumption at rest using indirect calorimetry, where energy consumption is determined by measuring the air the subject breathes.
2. Measurement of body composition, i.e., the proportion of body fat mass and lean mass, employing air displacement plethysmography. In addition, body circumference (e.g., waist and hips) and body skin folds (e.g., on the arm and waist) are measured.
3. Measurement of physical activity and sleep behavior under everyday conditions using accelerometry. The study participants are asked to wear the accelerometer in the form of a wristwatch continuously for 7 days.

#### Genome-Wide Association Study Analysis

We will perform a genome-wide association study analysis in manifesting vs. non-manifesting carriers aimed at discovering genetic modifiers of penetrance of *LRRK2* pathogenic variants.

#### Toxicological Analyses of Household Dust

A broad non-target liquid chromatography high-resolution mass spectrometry (LC-HR-MS) screening will be performed on household dust samples collected by participants in vacuum bags [see (10)], with identification of potential toxicants of interest via the *in silico* fragmenter MetFrag (11) coupled with open chemical databases such as PubChem (12) and CompTox (13), plus specific neurotoxicity-related candidate lists and end-point information (14).

## Results

A comprehensive eCRF has been developed, consisting of an investigator-rated (1 h) and a self-rated (1.5-h) part. The questionnaires are listed in Table 1. We used validated versions of questionnaires and scales in the local languages.

**Table 1:** Content of the LIPAD electronic case report form.

Demographic and general data	Genetic result, age, gender, years of education, the highest level of education, handedness, medication/treatment, medical history, family history concerning PD
PD criteria	MDS clinical diagnostic criteria for PD, absolute exclusion criteria, red flags
Clinical scores	MDS-UPDRS (Movement Disorder Society – Unified Parkinson's Disease Rating Scale) Hoehn & Yahr scale Schwab & England scale Brief smell identification test (BSIT) Montreal Cognitive Assessment (MoCA)
The self-rating part	PD risk factor questionnaire (PD-RFQ-U) Food frequency questionnaire Autonomic dysfunction (SCOPA-AUT) WHO Quality of life questionnaire (WHO-QoL) Pittsburgh Sleep Quality Inventory Epworth Sleepiness Scale Hospital Anxiety and Depression Scales

**Figure 2:**
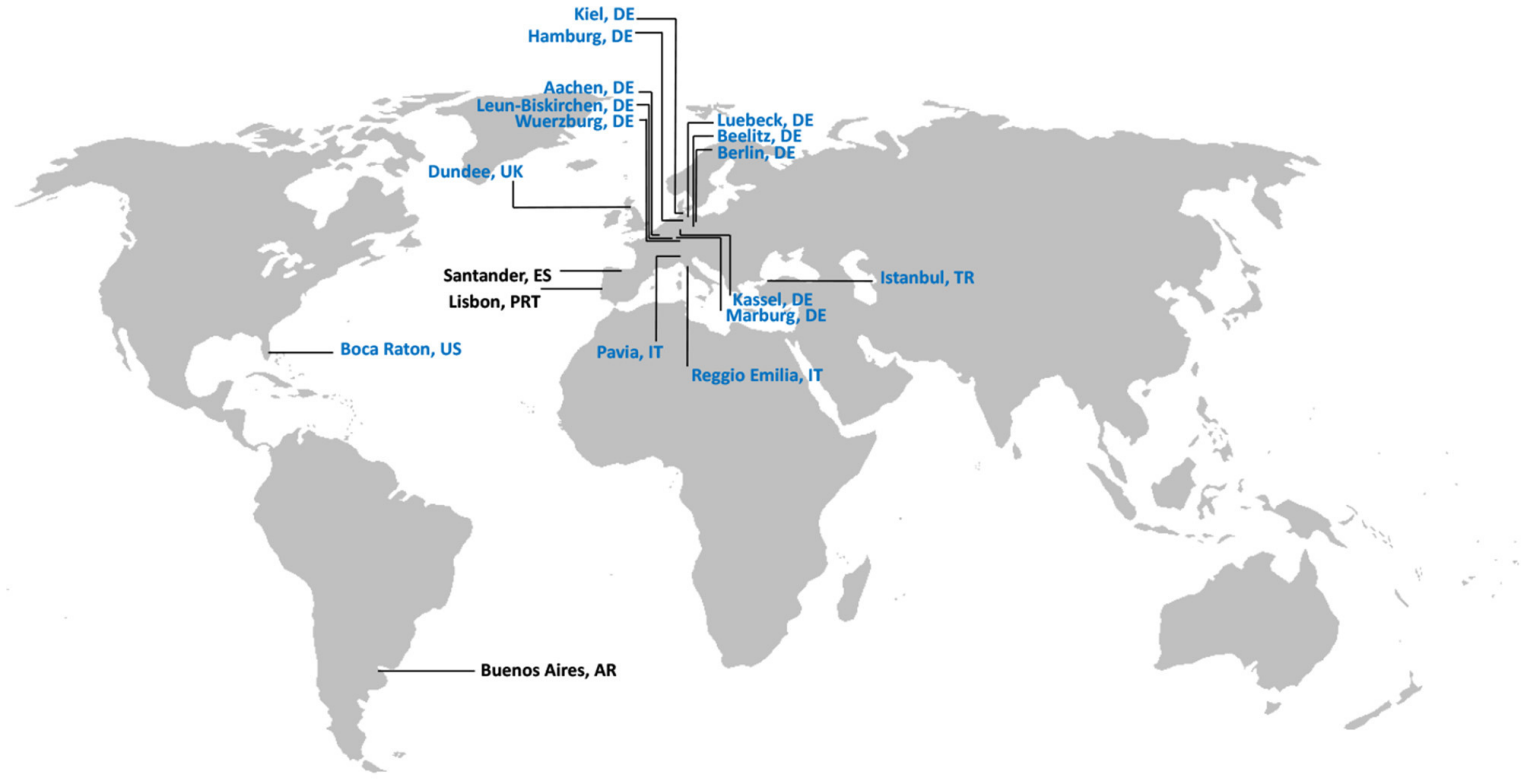
International centers participating in LIPAD. In blue: centers with ethics approval; in black: centers in the process of receiving ethics approval.

The first 10 centers across Germany and five international sites have been initiated with further centers currently undergoing ethical review (Figure 2). The first 150 participants have been enrolled (as of March 25th, 2021); Table 2.

**Table 2:** Exemplary demographic and clinical characteristics of the first 150 enrolled LIPAD participants.

	***LRRK2*** **+/PD+ (group 1)**	***LRRK2*** **+/PD-(group 2)**	**iPD;LRRK-/ PD+ (group 3)**	**genPD (group 4)**	**HC (group 5)**
	***N*** **= 7**	***N*** **= 1**	***N*** **= 115**	***N*** **= 25**	***N*** **= 2**
Sex (male), *n* (%)	2 (28.6%)	0	64 (55.7%)	15 (60%)	2 (100%)
Age, mean (±SD)	60.6 (± 9.2)	40	63.3 (± 10.8)	56.4 (± 11.6)	57 (± 12)
Family history of PD, *n* (%)	3 (42.9%)	1 (100%)	19 (16.5%)	7 (28%)	1 (50%)
MDS-UPDRSIII, mean	37.7 (± 20.3)	0	34.3 (± 14.4)	28.6 (± 12.5)	4 (± 4)
(±SD), *n* complete	*N* = 3	*N* = 1	*N* = 59	*N* = 11	*N* = 2
H&Y, mean (±SD)	3 (± 0.6)	0	2.1 (± 0.6)	2.1 (± 0.7)	0.5 (± 0.5)
Schwab & England Scale, mean (±SD)	71.7 (± 21.1)	-	81.9 (± 14.7)	84.4 (± 11.2)	100 (± 0)
MoCA, mean (±SD)	25.8 (± 3.3)	29	26.6 (± 3.3)	27.6 (± 2.1)	26.5 (± 1.5)
BSIT, mean (±SD)	6.2 (± 1.7)	11	6 (± 2.6)	6.5 (± 3.3)	11 (± 1)

*H&Y, Hoehn and Yahr; MoCA, Montreal Cognitive Assessment; BSIT, Brief Smell Identification Test*.

## Discussion

LIPAD is a large-scale international scientific effort focusing on deep phenotyping of *LRRK2*-linked PD and healthy pathogenic variant carriers, including a comparison with additional relatively frequent genetic forms of PD, and with a future perspective to identify genetic and environmental modifiers of penetrance and expressivity.

The clinical assessment in LIPAD is close to the one used in the currently largest multinational cohort of genetic PD (PPMI), except for lack of collection of cerebrospinal fluid, but with the addition of an extensive questionnaire on environmental factors (15). Further, there are no restrictions on the age at diagnosis or medication for inclusion in the LIPAD study. Several other multinational prospective studies focusing on pathogenic *LRRK2* variants included only a small number of populations (4, 16, 17), revealing an important knowledge gap in multinational, standardized, genetic prospective studies, which LIPAD is aiming to close.

A comprehensive investigation of modifiers of penetrance and expressivity requires assessment of a considerable number of pathogenic variant carriers. Due to the direct enrolment of family members at an international scale, the LIPAD study will enable the analysis of modifiers of penetrance in different populations in one setting. Our recruitment strategy, mainly through the international multicenter ROPAD study, will ensure participation of a large number of *LRRK2*+/PD+ patients.

With the nested studies, which will be conducted on a subset of participants, we are aiming to provide a more in-depth phenotypic characterization especially of non-affected carriers of *LRRK2* pathogenic variants.

Currently, a complication affecting many patient studies across the world are measures imposed on personal visits to contain SARS-CoV-2 in many countries, limiting the possibility for the collection of data and biomaterials. To allow the study to proceed under these circumstances, we adjusted the protocol and procedures to collect the necessary data online. After making an online appointment, patients receive a package with patient information, informed consent forms, and a smell test by post. Then an online appointment with the study team takes place, during which the study is explained, the data is collected and the examination is performed. Study participants send signed informed consent forms and a household dust sample back to the study center by post. Biomaterials will be taken at a general practitioner's office or the study center at a later time point when study visits become possible again. The study will revert to the on-site recruitment as soon as possible after the pandemic restrictions are lifted.

LIPAD aims for a longitudinal follow-up of its study participants over a 10–15-year time period. The exact protocol is currently being developed and potential funding opportunities are being explored.

## Ethics Statement

The studies involving human participants were reviewed and approved by Ethics Committee (EC) of the University of Lübeck (19-065); EC LÄK Hessen (2019-1364-zvBO); EC LÄK Hamburg (MC-002/20); EC LÄK Brandenburg [AS 35(bB)/2020]; EC Würzburg (161/19_z-sc); EC Kiel (B 292/19); EC Marburg (111/20); EC UK IRAS (project ID: 275553, REC reference: 20/NE/011); EC USA IRB tracking number: 20193494; Pavia, Italy (20200048346); Reggio Emilia, Italy (1268/2020/OSS/AUSLRE); Istanbul, Turkey (2020.362.IRB1.144). The patients/participants provided their written informed consent to participate in this study.

## Lipad Study Group Investigators (in Alphabetical Order)

Daniela Berg, University Hospital Schleswig-Holstein, Kiel, Germany; Leonor Correia Guedes, Hospital de Santa Maria, Lisbon, Portugal; Ilona Csoti, Natalia Koleva-Alazeh, Gertrudis-Kliniken im Parkinson-Zentrum, Leun-Biskirchen, Germany; Georg Ebersbach, Doreen Gruber, Neurologisches Fachkrankenhaus für Bewegungsstörungen/Parkinson, Beelitz-Heilstätten, Germany; Sibel Ertan, Özgür Öztop Çakmak, Department of Neurology, Koç University School of Medicine, Istanbul, Turkey; Jon Infante, University Hospital Marqués de Valdecilla, Santander, Spain; Stuart Isaacson, Parkinson's Disease and Movement Disorder Center of Boca Raton, Boca Raton, U.S.A.; Christine Klein, Universität zu Lübeck, Lübeck, Germany; Andrea A. Kühn, Friederike Borngräber, Charité University Medicine Berlin, Berlin, Germany; Marcello Merello, Malco Rossi, FLENI Foundation, Buenos Aires, Argentina; Brit Mollenhauer, Paracelsus-Elena-Klinik, Kassel, Germany; David J. Pedrosa, University Hospital Marburg, Marburg, Germany; Kathrin Reetz, University Hospital RWTH Aachen, Aachen, Germany; Esther Sammler, University of Dundee, Dundee, Scotland, UK; Enza Maria Valente, Department of Molecular Medicine, University of Pavia and IRCCS Mondino Foundation, Pavia, Italy; Micol Avenali, IRCCS Mondino Foundation, Neurorehabilitation Unit, Department of Brain and Behavioral Sciences, University of Pavia, Pavia, Italy; Franco Valzania, Giulia Toschi, Francesco Cavallieri, Azienda USL – IRCCS, Reggio Emilia, Italy; Jens Volkmann, University Hospital Würzburg, Würzburg, Germany; Simone Zittel, Lisa Prilop, University Medical Center Hamburg-Eppendorf, Hamburg, Germany.

## Author Contributions

TU: organization and execution of the research project, design and execution of statistical analysis, and writing the first draft of the manuscript. E-JV and AR: conception and organization of the research project, review and critique of statistical analysis, and review and critique of the manuscript. NS and SS: organization and execution of the research project, review and critique of statistical analysis, and review and critique of the manuscript. VS, XB, HG, TF, AH, NK-A, IC, DG, ESa, EV, FV, SZ, AB, SE, and GG: organization of the research project, review and critique of statistical analysis, and review and critique of the manuscript. PB, AG, ESc, JT, DB, SI, AK, BM, DP, and JV: review and critique of statistical analysis and review and critique of the manuscript. KL: organization of the research project and review and critique of the manuscript. NB, MK, and CK: conception and organization of the research project, design and review and critique of statistical analysis, and review and critique of the manuscript. All authors contributed to the article and approved the submitted version.

## Conflict of Interest

VS, XB, HG, TF, AH, PB, and AR were employed by company CENTOGENE GmbH. The authors declare that this study received funding from Centogene GmbH. The funder was involved in the study design, organization of the research project and review and critique of the manuscript.

## Publisher's Note

All claims expressed in this article are solely those of the authors and do not necessarily represent those of their affiliated organizations, or those of the publisher, the editors and the reviewers. Any product that may be evaluated in this article, or claim that may be made by its manufacturer, is not guaranteed or endorsed by the publisher.
