# Supplementary material for: LIPAD (LRRK2/Luebeck International Parkinson's Disease) Study Protocol: Deep Phenotyping of an International Genetic Cohort
Source: Front Neurol. 2021 Aug 9;12:710572. doi: 10.3389/fneur.2021.710572 (PMC8406937; doi:10.3389/fneur.2021.710572)
Supplement: Supplementary file 1 [file Data_Sheet_1.docx]

**Supplementary material**

**Methods S1. Approval by institutional review boards.**

The Lübeck/*LRRK2* International Parkinson’s Disease Study (LIPAD) has been approved by the following local and central Institutional Review Boards and Ethics Committees: EC Lübeck: 19-065; EC LÄK Hessen: 2019-1364-zvBO; EC LÄK Hamburg: MC-002/20; EC LÄK Brandenburg: AS 35(bB)/2020; EC Würzburg: 161/19_z-sc; EC Kiel: B 292/19; EC Marburg: 111/20; EC UK IRAS project ID: 275553, REC reference: 20/NE/011; EC USA IRB tracking number: 20193494; Pavia, Italy: 20200048346; Reggio Emilia, Italy: 1268/2020/OSS/AUSLRE; Istanbul, Turkey: 2020.362.IRB1.144.

**Methods S2. The three levels of biomaterial collection.**

Level 1:

Description: Blood samples for DNA extraction to be sent to Luebeck as soon as possible within two weeks, no processing at the site.

Target group: Centers without research laboratory to process samples.

Requirements: Clinical investigation according to the LIPAD eCRF and additional blood sample: 3 EDTA 5 ml tubes.

Level 2:

Description: Level 1 plus collection of PaxGene tubes for the analysis of RNA and dust sample for toxicological analysis. Blood samples do not need processing at the site but need to be sent to the study site in Luebeck the same day.

Target group: Majority of centers, own laboratory is not required, the samples should be stored at room temperature or refrigerated (at 4 degrees Celsius) and sent to the closest Level 3 center or Institute of Neurogenetics in Luebeck on the same day.

Requirements: eCRF and biosample collection as on Level 1, additional blood sample in PAXgene tubes for RNA extraction and dust sample from the household vacuum cleaner for toxicological analysis.

Level 3:

Description: Level 2 plus collection of serum/plasma samples for the analysis of metabolites; urine for analysis of proteins, NGS-based sequencing of the mitochondrial genome and search for mitochondrial DNA deletions. Processing on site is necessary within 1 hour after collection of the blood samples.

Target group: Institute of Neurogenetics (University of Luebeck) and at least 1 Level 3 center per country (usually academic centers equipped with lab).

Requirements: eCRF and biosample collection as on level 1 and 2, collection of additional tubes for plasma and serum extractions, urine sample.
